# Faculty perspectives on open educational resources: A case study of a Hong Kong Higher Education Institution

**DOI:** 10.1371/journal.pone.0349147

**Published:** 2026-08-03

**Authors:** Chenggui Duan, Billy Tak Ming Wong

**Affiliations:** 1 School of Information Technology in Education, South China Normal University, Guangzhou, China; 2 Li Ka Shing School of Professional and Continuing Education, Hong Kong Metropolitan University, Hong Kong SAR, China; 3 Institute for Research in Open and Innovative Education, Hong Kong Metropolitan University, Hong Kong, Hong Kong SAR, China; University of Cape Town, SOUTH AFRICA

## Abstract

The global move toward open education has led to greater receptivity to the concept of Open Educational Resources (OER) in terms of being able to foster inclusivity and innovation. However, it remains challenging to implement OER in the institutional context. This study examined teachers’ perceptions on the adoption, challenges and future of OER at a Hong Kong higher education institution. A qualitative interpretivist approach was taken, and eight faculty members across different disciplines participated in semi-structured interviews. The major findings of this study are as follow: First, there is a distinct conceptual gap where faculty equate “free” commercial tools with “open” licensed resources; adoption is driven by “pedagogical pragmatism” rather than an ideological commitment to openness. Second, significant barriers persist, including the time burden of curation, quality concerns, and a lack of institutional incentives. Third, the study highlights the disruptive potential of Artificial Intelligence (AI) on open education. Participants view AI not merely as a tool but as a future “co-creator” that could automate content generation. These findings mean the shift for faculty is becoming a facilitator and course designer not just a content disseminator. The study also suggests that institutional policy must move beyond basic technical support to provide holistic professional development focused on copyright literacy, open licensing, and the integration of AI within open educational ecosystems.

## Introduction

The global higher education landscape changes in an unprecedented period and digital technologies develop as the Open Movement expands, which advocates for greater openness in education [1,2]. Open Educational Resources (OER) and Open Educational Practices (OEP) are central to the Open Movement. OER are defined as learning, teaching, and research materials in any format and medium that reside in the public domain or are under copyright that have been released under an open license, that permit no-cost access, re-use, re-purpose, adaptation, and redistribution by others [3]. OEP involves the collaborative and innovative use of these resources to create participatory learning cultures [4].

It is important to distinguish such “open” concepts from “free” (libre) resources. While many resources are “free” (gratis) to use, like Kahoot, Google Forms, and others, they do not grant the “libre” rights to modify, remix and redistribute that are central to the notion of openness as currently understood. This study takes a broad view of faculty use of open pedagogy, going beyond openly licensed resources to examine access and use of the larger ecosystem of digital resources and tools that support open pedagogy.

The development of OER in Hong Kong offers a unique case study of technological potential versus practical implementation. According to Li et al., Hong Kong’s education sector is well-equipped with advanced infrastructure, yet the uptake of OER in Hong Kong has been historically “sluggish” and conservative [5,6]. These studies argue that the challenges are not technical but attitudinal, with educators showing reluctance to share materials and students displaying limited understanding of open licenses. Furthermore, institutions like the Hong Kong Metropolitan University (formerly The Open University of Hong Kong) have pioneered efforts in defining and categorizing local resources—ranging from open textbooks to open courseware—to bridge these gaps [7]. And while studies have assessed the “readiness” of students and the professional development needs of faculty regarding educational technology generally [8,9], there remains a limited understanding of the qualitative, lived experiences of educators who are attempting to integrate these open practices into their daily teaching.

Despite the compelling benefits for OER, including cost savings, greater access to more improving learning experiences and consistent provision of 21st century skills [10], the challenge of integrating OER into the everyday curriculum of university teaching and learning remains complex and multidimensional. Teachers need to become the mediators of educational innovations for successful implementation [11–13]. However, their engagement with OER is shaped by a complex interplay of factors, including personal beliefs, pedagogical orientations, digital competencies, institutional policies, and perceived barriers [14]. For better informing of a contextualised response to OER adoption, it is important to have a clear understanding of teachers’ perspectives towards the use of OER.

A substantial body of international research has examined OER adoption and dissemination in the higher education sector, particularly its institutionalisation, barriers to finding resources, quality and copyright enforcement [13,15]. Yet there is little understanding of how educators at the university level are interacting with the broad definition of OER in the increasingly diverse post-secondary education landscape, especially in a specific context such as Hong Kong. Existing literature is often focused on individual tools or singular adoptions of OER, and little is known about how open technologies/pedagogies are conceptualised, appropriated and constrained by local educators in the globalised but culturally different higher education system of Hong Kong [16]. Recognizing that findings from one context cannot be universally applied to all Hong Kong universities, this research adopts a case study approach to explore the nuanced experiences of educators within a specific institutional setting.

The primary aim of this study is to investigate the perceptions of Hong Kong university faculty members toward OER implementation, including challenges and future implementation. The study seeks to go beyond measuring the level of OER adoption and to explore the motivations, experiences, and contextual factors that influence the faculty’s use of OER in higher education in Hong Kong.

To this end, the study attempts to answer the following research questions:

1. How do university teachers in Hong Kong perceive and define OER, and how do they distinguish between “free” and “open” resources in their practice?
2. How do they practice in adopting, adapting, and using various types of OER components (resources, tools, platforms, pedagogies)?
3. What are the perceived benefits and opportunities offered through OER to their teaching and professional practice, and to their students’ learning and development?
4. What important individual, pedagogical, institutional, technical, and cultural barriers do these people face as they try to implement OER?
5. How do they see their roles develop in an increasingly open learning environment through technological mediation and which types of institutional, collegial, technical and pedagogical supports would faculty members like receiving to support their integration of OER effectively?
6. What are their views on the future development of OER within the Hong Kong higher education landscape?

This study aims to fill a critical gap in understanding the experiences and perspectives of university teachers in Hong Kong regarding the adoption and integration of OER. By exploring their beliefs, practices, and challenges, this research not only addresses local needs but also contributes to the global movement toward openness in education, ensuring that technological advancements translate into meaningful and equitable educational outcomes.

## Methods

The study used qualitative research within the interpretivist model. Interpretivism assumes that reality is socially constructed and therefore, to understand human social realities, it is important to understand the meanings that people attach to those realities [17]. Such an approach is thus particularly suitable for capturing the contextually and subjectively diverse views and lived experiences of Hong Kong university teachers towards the adoption of OER in-depth.

### Research design: Reflexive thematic analysis

Qualitative data were analyzed using reflexive thematic analysis (TA) techniques outlined by Braun and Clarke [18,19]. TA is a theoretically flexible and rigorous approach for identifying, analyzing, and interpreting themes within qualitative data. TA was chosen because it can provide the rich, complex and detailed account of the data-set that closely matched the research questions [19]. The reflexive nature of TA emphasizes the active role of the researcher in constructing themes through interpretive engagement with the data rather than treating themes as pre-existing entities [13]. This approach involved continuous reflection on assumptions, analytical choices, and the researcher’s positionality throughout the process.

### Participant recruitment and sample

A purposive sampling strategy was used to recruit active faculty members from across departments and from various career stages in a university in Hong Kong to participate in the study. Potential participants were approached through university connections and email. Recruitment was carried out in Spring 2024. Eight participants provided informed consent to participate.

The sample represented various subject disciplines including nursing, computer science, education, engineering, and social sciences and business. The participants also had a range of teaching experience. P1 to P8 were the pseudonyms used for the respondents participating in the study. In total, the interviews lasted approximately 503 minutes. Participant characteristics and teaching contexts are presented in Table 1.

**Table 1:** Participant profile.

Pseudonym	Gender	Discipline
P1	Male	Clinic nursing
P2	Female	Materials science
P3	Male	Accounting
P4	Male	Translation
P5	Male	Electronic Circuits
P6	Male	Education technology
P7	Female	Early childhood education
P8	Female	Health sciences

### Data collection instrument and procedure

Data were collected using semi-structured interviews guided by a protocol developed from a literature review on OER adoption, faculty development, and technology integration in higher education. The interview guide included open-ended questions designed to elicit detailed responses about participants’:

- Definitions and perceptions of OER;
- Examples of OER use (resources, tools, and practices);
- Perceived benefits and drawbacks of OER;
- Institutional support received or needed;
- Challenges encountered in implementing OER;
- Perceived impact of OER on their teaching roles;
- Future outlook on OER within higher education.

Probes were used flexibly to explore emergent topics and deepen understanding. Interviews were conducted face-to-face during the 2024 Summer, with participants’ explicit consent. All interviews were audio-recorded and subsequently transcribed verbatim, producing a dataset of approximately 64,408 words.

### Data analysis process

All eight transcripts were iteratively analyzed using Braun and Clarke’s six-phase reflexive thematic analysis guidance [18,19]. This approach was chosen due to its flexibility and capacity to provide a rich, detailed and complex account of the data, whilst allowing researchers to stay close to the voice of participants. S1 File contains the data underpinning the thematic analysis reported in this article.

#### 1. Familiarization with the Data.

The first part of the analysis process involved the researchers reading and re-reading the eight interview transcripts to understand and get an overview of the participants’ experiences. The analysis cycle began with active reading and re-reading of transcribed data to note down initial ideas and identify patterns to give a sense of the data.

#### 2. Generating initial codes.

The entire data set was regularly coded in an inductive manner, in which the features of the data which seemed interesting or relevant to the research questions were identified and labeled. The first was thorough data-driven initial coding, whereby the researchers were open to any category that may arise without the influence of existing theory and in consideration of all data. This phase of coding resulted in the generation of 60 codes (see Table 2), with representation across all eight transcripts.

**Table 2:** Initial codes extracted from interview data (Excerpt).

Code ID	Initial Code	Description	Representative Quote	Participant(s)
IC01	Free tool preference	Preference for free over paid software	“We are looking for some free (software) to give them.”	P7, P5, P8
IC02	Cost-driven adoption	Using free tools due to budget constraints	“Because of no money” (referring to using Linux)	P7, P5
…	…	…	…	…
IC60	Funding mechanism complexity	Complex procedures for obtaining funding	“Receiving money through QEM involves a relatively complex process and has high requirements.”	P1

#### 3. Generating initial themes.

The various codes were sorted and collated into potential themes. The researchers analyzed the relationships between codes, between themes, and between different levels of themes. During the interpretive phase, codes with similar conceptual ideas were grouped together, resulting in the identification of 18 candidate themes, an overview of which is presented in Table 3. The analysis moved from a descriptive to a conceptual level.

**Table 3:** Initial themes (Excerpt).

Initial theme ID	Initial theme	Related codes	Primary participants
IT01	Understanding of “Free” vs “Open”	IC01, IC02, IC03, IC04, IC55	P4, P3, P7, P8
IT02	Limited OEP Awareness	IC05, IC24, IC52	P3, P4, P8, P1, P2
…	…	…	…
IT18	Student Skill Development	IC42, IC52	P1, P2, P3, P5

#### 4. Developing and reviewing themes.

Themes were examined in relation to coded extracts and the entire data set to determine whether they made sense and worked in relation to the research questions. The themes were merged, split or discarded based on their clarity, distinctiveness and contribution to the analysis.

#### 5. Defining and Naming Themes.

The definition of each theme articulated its general meaning and scope, including its boundaries. A brief, descriptive name to express its meaning was developed for each theme. This ensured consistency in its use at each stage of the analysis. Six overarching themes were developed that captured the total data set.

#### 6. Producing the report.

The final stage focused on selecting excerpts from participants’ interviews for each theme and writing an analysis for the Results section, where the story of the data was described concerning the research questions.

Throughout the analysis, reflexivity was maintained through the notes taken during the analysis, continual reflective analysis of decisions made during interpretation. To enhance rigor, initial coding and theme development were discussed with a research assistant to ensure interpretive credibility.

## Results

The reflexive thematic analysis of interviews with eight university teachers (P1–P8) in Hong Kong identified six interconnected core themes, which provide a comprehensive understanding of teachers’ perspectives and practices related to OER.

### Theme 1: Diverse Understandings and Perceptions of OER: A Spectrum from ‘Free’ to ‘Open’

In the initial phase of the study, there was a lack of consensus and clear understanding of the definition and scope of OER. Respondents supported the notion of free access to educational resources. However, the underlying open licenses for modification and co-creation of OER and similar Open-Source Software (OSS) were not perceived well. It includes the whole range between a realistic view of availability of free of charge tools and a more philosophical view of openness itself as a value.

**Pragmatic Views of** “**Free**” **Tools**: At the opposite end of the spectrum, a couple of participants used OER to mean free online tools or resources like Google, free to use in the sense that they do not cost anything. For example, asked what online technologies they used to support their teaching, P6 replied, “We use the most basic ones like Kahoot, Edpuzzle, and Nearpod... basically all of them are free tools.” Further on in the interview, they said they were aware of the difference between ‘free’ and ‘open’ but used them because one can “register with an email and use them.” P7 also started his interview with the most basic tools, and didn’t talk about niche open-source software. “The easiest one, which doesn’t cost money and is accessible... is Google. Teaching students to use Google Forms is the easiest way to collect data.” For them, being ‘free to use’ was far more important than adherence to a definition of open licensing.

**Subtle Understanding of Openness**: The difference between “free” and “open” became more subtle along the spectrum. P6 argued that they are “two different concepts.” “Free tools might offer certain functions... but they often come with many restrictions. In that sense, they are not as ‘open’ as they appear. Open educational resources... imply the potential to be open for access or modification, meaning they provide the source code for you to modify.” P5 explained this distinction, based on his engineering education, as a separation between, for example, open-access information, such as “open textbooks from Rice University,” and OSS. He wanted “GNU software” to have alternatives to commercial licenses which are not free software. Students could install the software on their computers without limits or charges. The group identified these licenses, which included the right to access, amend, and redistribute software, with formal definitions of freedom [3].

**Limited Knowledge of OEP**: There is limited awareness of practices associated with OEP, such as students being involved in the co-creation of open knowledge activities, and specific open pedagogies. Some teaching practices may align with OEP without being overtly identified as such (e.g., group projects using shared online environments). However, teaching designed around open pedagogical principles was generally not observed. This suggests that for some instructors, the adoption of tools and resources is more popular than the pedagogical shift that accompanies openness.

### Theme 2: Adoption patterns: Balancing commercial tools with open resources

Adoption strategies were identified that combined a “mixed economy” of tool use and a practical response rather than being strictly an open source or OER ethic. Perhaps the most consistent finding was for mixed faculty practice to favor commercial platforms for facilitation that are “free-to-use” and OER to meet content needs. This is less due to the license’s “openness” and more because of its utility for teaching and accessibility.

**Prevalence of “Free” over “Open” for Interaction:** The participants were reliant on free to use proprietary platforms, and they often mentioned Kahoot, Edpuzzle, and Nearpod as tools that kept learners engaged (P1, P6). P1 described using such tools when “Students are... shy or afraid to communicate. But they are willing to participate in Kahoot games.” In these cases, it was the “free” cost of such tools, rather than the open license, that provided the impetus to adopt the tool.

**The Role of Institutional Platforms:** Adoption was influenced by the institutional Learning Management System (LMS). This was the system of record for course delivery and, being a centralized system with a rigid structure, it was less effective at leveraging open resources. The contrast of P7’s experience with “5-credit distance learning courses [having] fixed layouts... making it difficult to integrate open resources” with full-college course pages likewise suggests that technical infrastructure has a large impact on OER use.

**Discipline-Specific Adoption of OER/OSS:** P5 described relying on OSS to overcome licensing limitations: “MatLab is a paid software... so we found a free alternative, GNU software.” P5 also described use of “open textbooks from Rice University” for students lacking foundational knowledge. There, the “open” functionality (free to modify and install) was the biggest attraction.

Pedagogical Drivers: Despite the economic aspect, the main reason was pedagogical. P8 wanted variety regarding pedagogical approaches: “A 4-hour lecture is too long... if it’s just me talking, the information is too singular. So I use YouTube videos to let others explain concepts with different styles.” P5 and P1 also cited the visualization of difficult concepts as a key factor. P5 felt that “Physics concepts like waves and diffraction are abstract... watching animations on YouTube makes them much easier to understand” while P1 used 3D apps and VR to help students visualize anatomical and physiological concepts that are difficult to interpret from 2D images in textbooks.

### Theme 3: Benefits and Opportunities: Enhancing Engagement, Access, Flexibility, and Collaboration

Other than resources used, participants mentioned advantages of OER in the context of pedagogy, and potential opportunities for collaboration and partnerships.

Enhanced Student Engagement and Active Learning: This was the most frequently cited benefit. P1 explained that “Students are often shy or afraid to communicate...but with Kahoot, they are willing to participate because it’s anonymous and game-based.” P6 reflected that the immediate feedback loops of platforms such as Edpuzzle “help students consolidate knowledge immediately” and enable students to monitor their understanding, in line with pedagogical theories supporting active learning and formative assessment [20].

**Increased Access to Diverse Resources:** The availability of OER was seen as a major advantage for supplementing curriculum gaps. P7 reported that she replaced physical visits to elderly care centers with online videos: “Since we couldn’t visit elderly centers due to the pandemic, I found YouTube videos about elderly care facilities to give students a concrete visual context.” Likewise, P5 argued that open textbooks from Rice University would support students without a background in Physics: “students without a background in the subject have access to high-quality reference materials without buying extra books.”

**Flexibility and Personalization:** OER was perceived to be a means of enabling flexibility and self-directed learning, which P8 was keen to develop: “I want to cultivate their self-learning ability. By being able to watch videos prior to class, students were able to digest content at their own pace while using class time for more in-depth discussion.” P6 also argued that Moodle’s modular structure allows for courses to be designed more flexibly, and that students can “experience the role of a teacher by designing their own online modules.”

Cost Savings: Although monetary concerns weren’t identified as a main motivator for creation, several respondents identified the lack of cost for many OER components as a major advantage. The most direct example of this response was from P5 who said “MatLab is very expensive commercial software. In my field we are doing the same simulation but using the GNU software, which is free and open-source. This is a huge savings for students.”

Collaboration and Innovation (especially with OSS/OEP): Some participants discussed pedagogical innovation through technology to solve a teaching problem. P2 described how they replaced physical chemistry labs with video demonstrations when their students could not be on campus during the pandemic: “We recorded the experiments but deliberately included mistakes... asking students to identify the errors.” Likewise, P7 described creating virtual learning materials with video editing software such as OpenShot when internship placements were not possible, showing how OER can reduce the impact of a disruption on teaching and learning continuity.

### Theme 4: Challenges and barriers: quality concerns, technical hurdles, time constraints, and institutional gaps

Despite the clear benefits of OER, challenges of quality, technology, teacher skills, and institutional support among others, could obstruct the adoption of OER.

OER Quality, Discovery, and Contextualization: Several participants noted that the variable quality of OER and the challenge of discovering them was a barrier. P1 described his concerns about quality, “We worry the quality of free tools might be worse... usually, we either find companies to develop for us or buy commercial software.” P1 described challenges finding appropriate material, “many online are not the type I want to teach... or might not be deep enough.” P5 described the time-consuming process of searching for appropriate OER: " The difficulty is that teachers have to find and screen [videos] themselves... try them one by one to see which explains clearly.” The discoverability and screening burden remains a key barrier [21].

**Technical Barriers and Support Deficits:** A major issue was the lack of support and maintenance on OSS. P6 noted that “Support is insufficient... If the server crashes, we have to solve it ourselves... basic issues are fine, but deep technical problems are difficult to handle without a strong technical team.” P6 was concerned that he had to rely on a small internal support team. Even her own hardware was incompatible, according to P7, “the notebook... didn’t support it... compatibility was a problem,” and she had to return to commercial software when her efforts to install an open-source operating system failed.

**Time Constraints and Workload:** Time required to develop and adopt OER worsened other academic workload. P1 did not refuse to use OER but said “we need to balance teaching, research and administrative duties”; and that developing or adopting OERs “takes a lot of time... time and manpower are problems.” According to P2, “Teachers need time and sufficient resources,” and “finding suitable resources requires significant investment from the teacher.”

Institutional and Systemic Factors: Many participants identified a lack of institutional incentives. P5 commented, “Teaching won’t get you promoted... only publishing papers and getting research grants help with promotion.” P7 reported that the university supports open learning, but said, “The concepts are not concrete... leaving teachers to fumble on their own.” P8 expressed concerns about intellectual property and data privacy in open educational tools, saying, “Students might not protect your intellectual property... they might upload materials to the cloud.”

**Digital Literacy and Confidence:** Some employees were tech-savvy but others alluded to different levels of digital literacy across staff. P1 said: “For some older teachers... they might not want to change or learn new things... it is difficult for them.” P4 explained: “Teachers have pressure... if you are not familiar with the new tools... and students ask you.”

### Theme 5: Evolving teacher roles and professional development needs: Shifting towards facilitation and design

The integration of OER prompted significant reflection on the changing nature of the teacher’s role in higher education.

**Shift from Transmitter to Facilitator/Designer:** There was broad agreement that technology such as OER would require breaking away from the ‘sage on the stage’ model. P2 described their role as, " My role is not singular... I am like a tour guide on a bus, leading passengers to different spots along the way... a navigator... constantly helping and answering questions,” rather than being the source of information. P5 added the dimension of curation: “The difficulty is that teachers have to find and screen resources... try videos one by one to see which explains clearly” (i.e., teachers act as filters). P2 described skills associated with critical literacy, that students need to learn “how to analyze the answer” provided by tools like AI and “judge if it is fake.”

**Need for Holistic Professional Development:** This shifts the role to a more expert mode and requires additional professional development beyond technical skills. P6 argued that it was necessary to explain “what open pedagogy is... and how it compares to traditional methods... what are the benefits?,” and P2 stressed discipline-specific relevance, explaining that the “themes are sometimes distant from my work... different disciplines are very different.” P8 stressed the “rapid pace of technological change” that teachers may find hard to keep track of without institutional support, such as lists of useful resources.

**Valuing Peer Learning and Support:** Beyond formal training, participants valued informal learning and support networks. P1 expressed a desire to act as an “advocate,” stating that after verifying a tool is good, one should “share it with colleagues to maximize the benefit.” Timely and accessible technical support was also deemed crucial; P6 noted the difficulty of resolving deep technical issues without a strong support team, stating, “If the server crashes... we have to solve it ourselves.”

Participants described role modelling by colleagues as easing self-learning. P1 stated, one should serve as an “advocate” and, having confirmed the tool is good, “share it with colleagues to maximize the benefit.” Timely and accessible technical support was also deemed crucial; P6 noted the difficulty of resolving deep technical issues without a strong support team, stating, “If the server crashes... we have to solve it ourselves.”

### Theme 6: Expectations and outlook for future OER development: Calls for quality, integration, policy, and community

Looking forward, participants expressed a mix of optimism about OER’s potential and clear expectations for improvements needed to realize that potential.

**Improved Resource Quality and Curation:** A strong desire emerged for mechanisms to ensure the quality and relevance of OER. One participant hoped the school would provide a summary or list. P8 stated, “Perhaps the university could provide a summary or list... of what open-source software is available... because filtering is very time-consuming.” P6 saw a “database to integrate open resources” in addition to local resources.

Enhanced Technology Usability and Integration: Expectations included more user-friendly interfaces and stability. P4 stated that “The most important thing is the user interface... it must be simple for the user,” while P6 said that “The interface is often the hardest part... open-source software often has design issues and isn’t very polished,” so users have to do more work to solve problems.

Stronger Institutional Strategy and Support: Clearer institutional support and strategy were seen as necessary: “The university needs to explain more about what open learning is... the concepts are not concrete enough,” said P7. Financial support to teachers “without cumbersome administrative procedures” was requested by P2 to help them adopt the new resources in their teaching. Having a strong technical infrastructure was another important factor: P6 for example highlighted technical problems with unstable servers and a lack of support staff.

**Potential of AI Integration:** Conversely, the potential of AI as a co-creator of OER was appreciated: according to P5, “The future should be the integration with AI models... students might ask AI to generate explanations or content,” eliminating the need for teachers to manually generate content. P6 viewed AI as a potential source of innovation, with “Open educational practices linked with AI... might provide some innovative ideas.”

**Fostering Communities of Practice:** Building a network was seen as important. P6 described the “snowball” model for dissemination: “Start with a group of people... develop examples... and then do a good promotion to recommend it to more people”; that is, a community of practice could be built from that initial small base.

### Thematic map of teachers’ perspectives on OER

**Fig 1:**
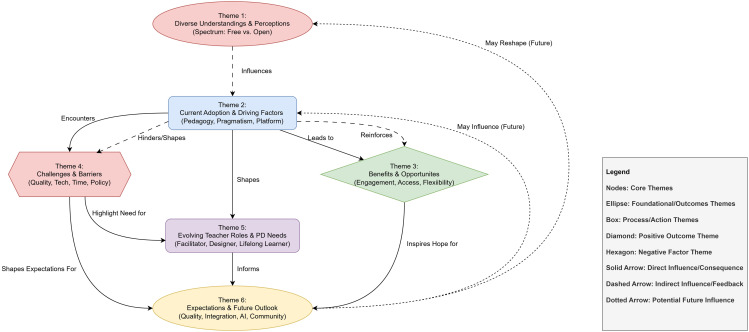
Thematic map of teachers’ perspectives on OER.

Fig 1 illustrates these six main themes, their inter-relationships, and factors that shape OER adoption, and the challenges and opportunities that Hong Kong university teachers envisage with OER. The shapes and arrows are described in the legend (figure caption) below.

The relationships depicted in the thematic map are grounded in recurring patterns and explicit connections identified during phases 4 and 5 of the reflexive thematic analysis. Below are some of the explanations of the key inter-theme relationships:

1. **Theme 1 (Diverse Understandings) indirectly influences Theme 2 (Adoption Patterns):** Teachers’ conceptualizations of OER (Theme 1) act as a cognitive framework shaping the scope and nature of technologies considered for adoption under Theme 2. For instance, participants who equated “open” with “free” tools tended to adopt platforms like Kahoot, while those with deeper understandings of OER principles sought openly licensed materials. This influence is depicted as ‘indirect’ because understanding itself doesn’t automatically trigger adoption, but it significantly shapes the scope and nature of the technologies teachers consider and are willing to try, acting as a cognitive filter or framework.
2. **Theme 3 (Benefits & Opportunities) positively influences Theme 2 (Adoption Patterns):** Adoption decisions were often motivated by perceived benefits, such as enhancing engagement or increasing flexibility, directly linking Theme 3 to Theme 2. For example, a teacher might adopt an open simulation tool because they believe it offers a unique interactive learning opportunity (Benefit) not available otherwise.

Table 4 provides a detailed summary of the key inter-theme relationships identified during the analysis.

**Table 4:** Key relationships in the thematic map.

Relationship	Description	Example
**Theme 1 → Theme 2** *(Indirect)*	Teachers’ understanding of OER shapes their adoption choices.	Those equating “open” with “free” tools adopt platforms like Kahoot.
**Theme 3 → Theme 2** *(Direct)*	Perceived benefits drive adoption of OER.	A teacher used Edpuzzle to boost student engagement.
**Theme 4 → Theme 2** *(Direct)*	Challenges hinder OER adoption.	Lack of time to find quality resources limits adoption.
**Theme 2 → Theme 5** *(Direct)*	Adoption of OER reshapes teachers’ roles and highlights new skill needs.	Using OER led a teacher to seek training on copyright and design.
**Theme 2 → Theme 6** *(Direct)*	Current adoption experiences inform future expectations.	Success with tools inspires optimism, while challenges lead to calls for better resources.
**Theme 4 → Theme 6** *(Direct)*	Challenge shapes expectations for the future.	Teachers want better support and curated OER to address current issues.
**Theme 5 → Theme 6** *(Direct)*	Reflections on roles and skills influence expectations for support and tools.	Teachers hope for training and platforms that align with their evolving roles.
**Theme 3 → Theme 6** *(Direct)*	Benefits fuel optimism for OER’s future.	Seeing flexibility in tools inspires hopes for more personalized learning environments.
**Theme 4 → Theme 5** *(Direct)*	Challenges highlight the need for new skills and support.	Struggling with technical issues shows the need for better digital training.
**Theme 6 → Theme 1** *(Potential)*	Future expectations may reshape how teachers understand OER.	Improved policies might expand teachers’ views of OER’s scope and potential.
**Theme 6 → Theme 2** *(Potential)*	Anticipated improvements may influence future adoption patterns.	Better support could encourage adoption, while pessimism could slow uptake.

## Discussions

This study outlines the perceptions of eight university teachers in Hong Kong regarding their knowledge about, use of, challenges to, and expectations for OER. The data were subjected to thematic analysis, resulting in six themes that characterize the context of OER in Hong Kong higher education institutions. This section discusses these findings in relation to the existing literature, considering similarities and differences as well as the implications for theory and practice.

### Diverse understandings: Navigating the terminology of openness

Participants’ understanding of OER ranged from knowing the difference between OER, OSS and Open Pedagogy, to ambiguity and conflation between “open” and “free.” This is in line with existing literature [22,23] that has shown “openness” to be context dependent and multi-faceted. While generally the sharing and access of OER for free was mentioned, the permission granted through an open license (e.g., the right to revision, remixing, etc., that are central to many definitions of OER, including UNESCO’s, 2019) or collaborative aspect of OSS or Open Pedagogy was less understood. For example, P1 and P7 gave free proprietary tools like Kahoot or Google Forms as an example of OER, indicating a practical approach of usability and cost rather than a strict adherence to open licensing. This is consistent with findings in other studies on motives for technology adoption (see, e.g., [24]).

### Adoption patterns: Pragmatism, pedagogy, and platform constraints

The commonest reasons for adopting OERs were pedagogical: student engagement (in P1, P6) and the availability of free resources (which depended the most on discipline or institutional infrastructure). The common use of interactivity via free online tools indicates a practical approach, whereby teachers’ endeavors are closely related to their available technology and efforts to engage students. That institutional LMS (open source or commercial, P4, P6, P7) are preferred for technology mediation shows the power of these institutional platforms. The infrequent use of OER and OSS (P1, P5) is likely due to the increased browsing, evaluation and reinvention effort needed (P1, P5) or need for technical knowledge (P6, P8). These concerns for OER adoption are widely reported in the international literature [21]. The motivators identified were: engagement, access to resources, flexibility, and cost. They were matched to technology adoption motivating factors in general frameworks such as UTAUT [24]. The choices of tools and resources (e.g., free tools, OER, OSS, etc.) were influenced by perceived usefulness, ease of use, availability of technical support, and teachers’ agency.

### Perceived benefits: Enhancing interaction, access, and flexibility

The value of OER in regards to student engagement, diverse perspectives, flexibility, and cost savings was discussed. OER benefits through interactive tools for classroom engagement (P1, P6) were also noted and are aligned with the research on active learning [20]. The sheer abundance and accessibility of OER (P5, P7) fit with one of the key rationales of open education, that of open access to knowledge (2, 11). The flexibility and adaptability of space, time and material afforded by OER (P1, P5) parallels the trend towards learner-centered and customized learning (1). Collaborating and creating with OSS (P8), as well as teachers becoming resource providers (P6), could signal the shift towards the transformative potential of open practices [22,23].

### Significant challenges: Quality, support, time, and systemic hurdles

The study highlighted the barriers to scaling up OER that are obvious in the wider literature, including challenges finding quality OER that are contextually relevant and culturally appropriate for their local contexts (P1, P5) [15]. The technical issues faced, especially around the lack of formal support and maintenance for OSS (such as Moodle (P6)), go beyond the hidden costs of “free” to the necessity of institutional technical structures in contexts where dedicated IT support for non-standard platforms is limited, or non-existent.

Commonly cited barriers were the pressure on teachers to find, evaluate, adapt and learn how to use OER effectively within already congested workloads (P1, P2), in line with research identifying teacher capacity and time as key to technology integration [25,26]. The lack of institutional policies, incentives and quality assurance checks (P5, P7) that could potentially support teachers also leads to a nebulous understanding of OER and reluctance to engage with or use them in greater depth. These findings resonate with others, noting the need for institutionalizing OER and systemic support [27]. Privacy and security of third-party tools is emerging as a key factor for institutions (P8).

### Evolving roles and development needs: From user to designer and facilitator

Indeed, the experience with OER aligns with teacher’s roles as facilitators, designers, curators and guides rather than providers (P2, P5, P6), and with constructivist or connectivist approaches and models, e.g., TPACK, that stress the interrelations among technological, pedagogical and content knowledge [28]. This will require wide-ranging professional development for staff, not only on technical issues, but also pedagogical aspects (P6) and assessment of OER (P5, P8). This may also include training on how to give back to the open ecosystem (P6). The need for peer collaboration and sharing (P1, P7) highlights the need for communities of practice to support teacher learning and technology adoption (OER) [29,30] which may be critical to stimulate and support teachers’ use of OER.

### Future outlook: Quality, integration, policy, and AI

Anticipated changes included quality and access to resources (P8), technology ease of use and integration (P4, P6), institutional and policy supports (P6, P7), and integration of artificial intelligence (P5, P6). There was also a move toward seeking better quality, curated, and potentially localized OER resources rather than simply free resources, suggesting a maturing attitude towards the concept of OER. Readiness to be integrated into an existing LMS is linked to interoperability and ease of use. The emphasis on policy and institutional support (including technical infrastructure and incentives) underscores the understanding that sustainable OER adoption requires systemic change, not just individual effort [31]. This cautionary optimism suggests a recognition of the potential of emergent technologies, and an openness to their application within open education. Building supportive communities (P6) in conjunction with sharing successful use cases were also seen as important strategies to encourage adoption.

In summary, the participants of this study recognize the possible benefits of using OER, but differing understandings, realistic concerns, important barriers (resource quality and technical/institutional support), and changing professional practices mean any successful and sustainable OER adoption will have to proceed on multiple fronts: resources, technology, policy, and professionals develop.

### Limitations

As a qualitative case study in a single university in Hong Kong, the research has some limitations. First, the sample size of eight is relatively small (n = 8) for the purpose of generalization. This research gave a full account of their experiences yet it does not apply to all Hong Kong universities or universities in other parts of Asia. The experiences of each faculty member would vary according to the university, their discipline, and the support services available to them at each institution.

Second, the present study was based on voluntary participation and thus possibly subject to self-selection bias. Those faculty members who volunteered to be interviewed for this research may have been more technology-wise and more education-oriented than average faculty members. Future research would benefit from larger quantitative studies to test the themes and concepts that emerged from the study across multiple institutions and a broader range of disciplines to provide a more representative picture of OER adoption in the region.

## Conclusion

This study explored eight teacher’s perceptions of OER in a higher education institution in Hong Kong, revealing a landscape defined by pedagogical pragmatism rather than ideological commitment. The findings indicate that while faculty members widely utilize “free” digital resources to enhance student engagement and reduce costs, there remains a conceptual ambiguity between “free” proprietary software and “open” licensed resources. Consequently, engagement with true OER and OSS remains context-dependent and constrained by the availability of institutional support.

The study confirms that the primary barriers to OER adoption are the time-intensive nature of locating quality resources, technical instability associated with OSS, and a lack of clear institutional incentives. These challenges are precipitating a fundamental shift in the educator’s role. Teachers are transitioning from traditional content disseminators to learning designers and curators. This shift requires them to navigate a complex ecosystem of digital resources, necessitating enhanced support in copyright literacy and technical troubleshooting.

This research also highlights the emerging impact of AI on the future of open education. Participants identified AI not merely as a technology or tool, but as a potential “co-creator” of content that could hugely alleviate the burden of resource generation. However, this integration brings new responsibilities: as AI lowers the barrier to creating content, the faculty’s role in critically evaluating, validating, and contextualizing AI-generated OER becomes increasingly important. The intersection of AI and OER represents a frontier that offers innovative possibilities for personalized learning but demands rigorous quality assurance.

In conclusion, this study suggests that sustainable OER adoption in Hong Kong higher education requires a multi-faceted approach. For instructors, professional development must include critical AI literacy and skills in evaluating open resources. For policymakers and institutions, the focus must shift from merely providing infrastructure to establishing comprehensive strategies that integrate OER policy with AI guidelines. By addressing the twin challenges of resource quality and technical support, and by proactively embracing the potential of AI, institutions can empower teachers to transform technological advancements into meaningful and equitable educational outcomes.

## Supporting information

S1 File Thematic analysis data: Open educational resources in higher education. (DOCX)
