# Supplementary material for: Faculty perspectives on open educational resources: A case study of a Hong Kong Higher Education Institution
Source: PLoS One. 2026 Aug 3;21(8):e0349147. doi: 10.1371/journal.pone.0349147 (PMC13432129; doi:10.1371/journal.pone.0349147)
Supplement: S1 File — (DOCX) [file pone.0349147.s001.docx]

Thematic Analysis Data: Open Educational Resources in Higher Education

# Overview

This document presents the complete thematic analysis process following Braun and Clarke's (2006) six-phase approach, based on semi-structured interviews with 8 university educators (P1-P8) regarding their perceptions and practices of Open Educational Resources (OER).

# Participant Information

| ID | Discipline | School |
| --- | --- | --- |
| P1 | Clinic nursing | School of Nursing and Health Studies |
| P2 | Materials science | School of Science and Technology |
| P3 | Accounting | School of Business and Administration |
| P4 | Translation/Language | School of Arts and Social Sciences |
| P5 | Electronic Circuits | School of Science and Technology |
| P6 | Education technology | School of Education and Languages |
| P7 | Early childhood education | School of Education and Languages |
| P8 | Health sciences | School of Nursing and Health Studies |

# Phase 1-2: Familiarization and Initial Coding

## Table 1: Initial Codes Extracted from Interview Data (Balanced Across All Participants)

| Code ID | Initial Code | Description | Representative Quote | Participant(s) |
| --- | --- | --- | --- | --- |
| IC01 | Free tool preference | Preference for free over paid software | "We need to find some free (software) for them." | P7, P5, P8 |
| IC02 | Cost-driven adoption | Using free tools due to budget constraints | "Because we have no money." (referring to using Linux) | P7, P5 |
| IC03 | Open vs free confusion | Conflating "open" with "free" | "Regarding what open educational resources are... I am not very clear about the definition." | P4, P3, P8 |
| IC04 | Open source awareness | Understanding of open source principles | "Open access or open modification... it can open the source code for you to modify." | P6, P5 |
| IC05 | OEP unfamiliarity | Limited understanding of Open Educational Practices | "I am not very clear, for students, how I can apply it in my classroom." | P8, P3, P4 |
| IC06 | YouTube as primary OER | Heavy reliance on YouTube for teaching | "I think the most commonly used by all of us is YouTube, playing videos." | P7, P1, P5, P8 |
| IC07 | Interactive tool usage | Using Kahoot, Edpuzzle, Nearpod | "Mainly the most basic Kahoot, as well as Edpuzzle and Nearpod." | P6, P1, P2 |
| IC08 | LMS centrality | Central role of learning management systems | "We have tried to teach students to use Moodle." | P6, P4, P3 |
| IC09 | Discipline-specific OSS | Using field-specific open source software | "Fortunately, MatLab has a free alternative... GNU software." | P5 |
| IC10 | Open textbook adoption | Using open textbooks | "Published by Rice University in the US... ask students to download it." | P5 |
| IC11 | Video editing tools | Using OpenShot for video editing | "I used OpenShot during the pandemic." | P7 |
| IC12 | Pedagogical motivation | Using OER for teaching improvement | "It may help them understand the learning content." | P5, P1, P2 |
| IC13 | Student engagement focus | Using tools to increase engagement | "It can help consolidate their knowledge... provide appropriate assessments in real-time." | P6, P1, P2 |
| IC14 | Visualization benefits | Using OER for concept visualization | "Some audiovisual media make it easier to understand these concepts." | P5, P1 |
| IC15 | Student background diversity | Addressing diverse student backgrounds | "Our students come from different backgrounds and have different understandings of the learning content." | P5, P2 |
| IC16 | Self-learning resources | Providing resources for student self-study | "We need to provide students with some self-learning resources." | P5, P4 |
| IC17 | Resource screening burden | Time spent filtering/selecting resources | "The difficulty is that teachers have to find and screen, try each video one by one." | P5, P2, P1 |
| IC18 | Resource instability | Resources disappearing or becoming unavailable | "Sometimes YouTube videos get removed... links become invalid." | P5 |
| IC19 | Technical support deficit | Lack of technical support for OSS | "Insufficient support... if our Moodle server crashes now, we have to solve the problem ourselves." | P6, P4 |
| IC20 | Platform limitations | LMS platform constraints | "It is difficult to use open teaching for a 5-credit distance course... the layout is all fixed." | P7 |
| IC21 | Time constraints | Limited time for learning new tools | "I am one of those very busy people, we really may not have that much time for training." | P7, P3, P4 |
| IC22 | Workload pressure | Heavy teaching workload limiting OER adoption | "Teachers already have a heavy workload, the teaching load is not small." | P7, P2, P4 |
| IC23 | Large class challenges | Difficulty implementing OER in large classes | "It is difficult to have discussions with four or five hundred people." | P8 |
| IC24 | Digital literacy gaps | Varying levels of digital competence | "Some colleagues did not even know how to use YouTube." | P7, P1 |
| IC25 | Self-taught approach | Learning OER tools independently | "Self-taught, and after self-learning, I still have to teach students." | P6, P1 |
| IC26 | Interface complexity | OSS interface less user-friendly | "I think the OpenShot interface is a bit complicated, the logic is not very clear." | P7, P8 |
| IC27 | Commercial vs OSS comparison | Comparing commercial and open source software | "Commercial software is more powerful in functionality, or easier to use." | P5, P2, P4 |
| IC28 | Role shift to facilitator | Teacher becoming guide/facilitator | "I am a facilitator, seeing what problems they have." | P7, P2 |
| IC29 | Resource introducer role | Teacher as resource curator/introducer | "First, teachers need to introduce these open educational resources to students." | P7, P1, P3 |
| IC30 | Continuous learning need | Teachers need ongoing professional development | "Teachers need to keep updating, otherwise they will be replaced by AI." | P5, P2, P4 |
| IC31 | Peer learning value | Learning from colleagues | "I work with (P6) next door every day researching how to use these software." | P7, P1 |
| IC32 | Training inadequacy | Insufficient formal training | "No training, but it was when I was helping teach anatomy that I started trying to use VR to teach." | P5, P1, P3 |
| IC33 | Policy support need | Need for institutional policy support | "University support, the most important is university support." | P7, P4, P1 |
| IC34 | Resource database request | Request for curated resource database | "It would be great to have such a list for everyone." | P8, P7 |
| IC35 | AI integration expectation | Expectation for AI in OER | "In the future, it should be integrated with AI models." | P5, P2, P4 |
| IC36 | Personalized learning vision | AI enabling personalized learning | "If students do not understand, they will ask the AI model... it is an interaction process between learners and AI." | P5 |
| IC37 | Community building need | Need for communities of practice | "After having that set of examples, there should be good promotion." | P6, P1 |
| IC38 | Quality assurance concern | Concerns about OER quality | "But the problem is the need to screen, because teachers need to watch and check those videos themselves." | P5, P1, P2 |
| IC39 | Copyright concerns | Worries about intellectual property | "I have a concern that this software is too open... it may not protect your intellectual property." | P8 |
| IC40 | Sustainability concerns | Worries about long-term availability | "It is open at this stage, but will it definitely remain open in the future?" | P8, P4 |
| IC41 | Openness as trend | Viewing openness as future direction | "The world should become more and more open." | P7, P1 |
| IC42 | Student skill advantage | Students with OER skills have advantages | "Definitely, in the future students need to understand that there is a lot of open source software." | P5, P3, P2 |
| IC43 | Reduced educational inequality | OER reducing educational disparities | "It can reduce educational inequality." | P8, P3 |
| IC44 | Diverse teaching formats | OER enabling varied teaching approaches | "My hope is that the formats can be more diversified." | P8, P2 |
| IC45 | Teacher as user | Teacher primarily as tool user | "I think I am a user." | P6, P1 |
| IC46 | VR/3D technology adoption | Using VR and 3D apps for teaching | "There is VR, MRA, and simulation... they also share what they have learned with other colleagues." | P1 |
| IC47 | Custom resource development | Developing own teaching resources | "Teaching resources we make ourselves are always most suitable for our courses; external ones do not quite meet our needs." | P1 |
| IC48 | Quality concerns about free resources | Worrying free resources may lack quality | "Free ones are rare because we worry that the quality of free teaching tools may be poor." | P1 |
| IC49 | Blended learning adoption | Using blended/hybrid learning approaches | "Blended learning mode, applied in one of the chemistry courses, dividing experiments into different parts to teach students." | P2 |
| IC50 | Student feedback importance | Valuing student feedback on OER | "The students' responses and feedback were very good. This result also moved us." | P2 |
| IC51 | Teacher as navigator | Teacher as guide/navigator for students | "I am a tour guide on a tour bus, leading passengers to different attractions." | P2 |
| IC52 | Information literacy concern | Concern about students' ability to evaluate information | "Searching for information online has both advantages and disadvantages; students may find incorrect information." | P2, P1 |
| IC53 | Assessment integration need | Need to integrate OER into assessment | "If you do not require them to use it in teaching and assess it, you are just teaching them to look." | P4 |
| IC54 | Practical necessity driver | Using tools only when practically necessary | "Free is not very important to us; if a tool is good or I need to use it, I am willing to pay." | P4 |
| IC55 | Concept confusion | Confusion between open learning and open resources | "We may have confused open resources with open learning." | P4, P3 |
| IC56 | Technology rapid change | Difficulty keeping up with fast-changing technology | "Technology is moving too fast; you may have just learned this software and others have stopped using it." | P4, P5, P8 |
| IC57 | Professional body constraints | Regulatory constraints on curriculum | "The medical council is quite conservative and does not encourage many new courses." | P1 |
| IC58 | Age-related adoption barriers | Older teachers struggling with new technology | "For some older teachers, I think they may not want to change or learn new things." | P1, P7 |
| IC59 | Student-teacher knowledge gap | Students sometimes more tech-savvy than teachers | "Sometimes some students are actually better than you." | P4, P8 |
| IC60 | Funding mechanism complexity | Complex procedures for obtaining funding | "Getting money through QEM involves quite complex procedures and high requirements." | P1 |

# Phase 3: Searching for Themes

## Table 2: Initial Themes (Grouping of Initial Codes)

| Initial Theme ID | Initial Theme | Related Codes | Primary Participants |
| --- | --- | --- | --- |
| IT01 | Understanding of "Free" vs "Open" | IC01, IC02, IC03, IC04, IC55 | P4, P3, P7, P8 |
| IT02 | Limited OEP Awareness | IC05, IC24, IC52 | P3, P4, P8, P1, P2 |
| IT03 | Video and Multimedia Resource Usage | IC06, IC11, IC14, IC46 | P1, P5, P7, P8 |
| IT04 | Interactive Tool Adoption | IC07, IC13, IC49 | P1, P2, P6 |
| IT05 | LMS as Central Platform | IC08, IC20 | P3, P4, P6, P7 |
| IT06 | Discipline-Specific Tool Selection | IC09, IC10, IC47 | P1, P5 |
| IT07 | Pedagogical Drivers | IC12, IC15, IC16, IC50 | P1, P2, P4, P5 |
| IT08 | Resource Discovery and Curation Challenges | IC17, IC18, IC38, IC48 | P1, P2, P5 |
| IT09 | Technical Support Gaps | IC19, IC26, IC27 | P2, P4, P5, P6, P7, P8 |
| IT10 | Time and Workload Barriers | IC21, IC22, IC23, IC60 | P1, P2, P3, P4, P7, P8 |
| IT11 | Digital Competence Variations | IC24, IC25, IC32, IC58, IC59 | P1, P4, P6, P7, P8 |
| IT12 | Institutional Support Needs | IC33, IC34, IC37, IC53, IC57 | P1, P4, P6, P7, P8 |
| IT13 | Evolving Teacher Roles | IC28, IC29, IC45, IC51 | P1, P2, P3, P6, P7 |
| IT14 | Professional Development Needs | IC30, IC31, IC32 | P1, P2, P4, P5, P7 |
| IT15 | AI and Future Technologies | IC35, IC36, IC56 | P2, P4, P5, P8 |
| IT16 | Quality and Sustainability Concerns | IC38, IC39, IC40, IC48 | P1, P4, P5, P8 |
| IT17 | Openness as Educational Trend | IC41, IC43, IC44 | P1, P2, P3, P7, P8 |
| IT18 | Student Skill Development | IC42, IC52 | P1, P2, P3, P5 |

# Phase 4: Reviewing Themes

## Table 3: Theme Refinement Process

| Initial Theme(s) | Refinement Action | Resulting Theme | Key Participants |
| --- | --- | --- | --- |
| IT01, IT02 | Merged - both relate to understanding of OER concepts | Theme 1: Diverse Understandings and Perceptions | P1, P2, P3, P4, P6, P7, P8 |
| IT03, IT04, IT05, IT06, IT07 | Merged - all relate to current adoption patterns | Theme 2: Current Adoption and Driving Factors | P1, P2, P3, P4, P5, P6, P7, P8 |
| IT17, IT18 | Merged with benefits from adoption themes | Theme 3: Benefits and Opportunities | P1, P2, P3, P5, P7, P8 |
| IT08, IT09, IT10, IT11, IT12, IT16 | Merged - all represent barriers | Theme 4: Challenges and Barriers | P1, P2, P3, P4, P5, P6, P7, P8 |
| IT13, IT14 | Merged - both relate to teacher development | Theme 5: Evolving Teacher Roles and PD Needs | P1, P2, P3, P4, P5, P6, P7 |
| IT15, IT12 (partial), IT17 (partial) | Merged - all relate to future expectations | Theme 6: Expectations and Outlook | P1, P2, P4, P5, P6, P7, P8 |

# Phase 5: Defining and Naming Themes

## Table 4: Final Themes with Sub-themes

| Theme | Theme Name | Sub-themes | Key Codes | Primary Participants |
| --- | --- | --- | --- | --- |
| Theme 1 | Diverse Understandings and Perceptions of OER: A Spectrum from 'Free' to 'Open' | 1.1 Pragmatic Views of "Free" Tools; 1.2 Nuanced Understanding of Openness; 1.3 Limited Awareness of OEP | IC01-IC05, IC24, IC55 | P1, P3, P4, P6, P7, P8 |
| Theme 2 | Adoption Patterns and Driving Factors: Pragmatism, Pedagogy, and Platform Influence | 2.1 Widespread Use of Interactive Free Tools; 2.2 Centrality of the LMS; 2.3 Discipline-Specific Adoption; 2.4 Key Drivers | IC06-IC16, IC46, IC47, IC49 | P1, P2, P3, P4, P5, P6, P7, P8 |
| Theme 3 | Benefits and Opportunities: Enhancing Engagement, Access, Flexibility, and Collaboration | 3.1 Enhanced Student Engagement; 3.2 Increased Access to Resources; 3.3 Flexibility and Personalization; 3.4 Cost Savings; 3.5 Collaboration and Innovation | IC12-IC16, IC41-IC44, IC50 | P1, P2, P3, P5, P7, P8 |
| Theme 4 | Challenges and Barriers: Quality Concerns, Technical Hurdles, Time Constraints, and Institutional Gaps | 4.1 OER Quality and Discovery; 4.2 Technical Barriers; 4.3 Time Constraints; 4.4 Institutional Factors; 4.5 Digital Literacy | IC17-IC27, IC38-IC40, IC48, IC52-IC54, IC56-IC60 | P1, P2, P3, P4, P5, P6, P7, P8 |
| Theme 5 | Evolving Teacher Roles and Professional Development Needs: Shifting Towards Facilitation and Design | 5.1 Shift to Facilitator/Designer; 5.2 Need for Holistic PD; 5.3 Valuing Peer Learning | IC28-IC32, IC45, IC51 | P1, P2, P3, P4, P5, P6, P7 |
| Theme 6 | Expectations and Outlook for Future OER Development: Calls for Quality, Integration, Policy, and Community | 6.1 Improved Resource Quality; 6.2 Enhanced Technology Usability; 6.3 Stronger Institutional Strategy; 6.4 AI Integration Potential; 6.5 Communities of Practice | IC33-IC37, IC41, IC53 | P1, P2, P4, P5, P6, P7, P8 |

## Table 6: Participant Contribution Summary

| Participant | Total Codes Contributed | Primary Themes Contributed |
| --- | --- | --- |
| P1 | 18 | Themes 1, 2, 3, 4, 5, 6 |
| P2 | 15 | Themes 2, 3, 4, 5, 6 |
| P3 | 8 | Themes 1, 2, 3, 4, 5 |
| P4 | 14 | Themes 1, 2, 4, 5, 6 |
| P5 | 16 | Themes 2, 3, 4, 5, 6 |
| P6 | 12 | Themes 1, 2, 4, 5, 6 |
| P7 | 14 | Themes 1, 2, 3, 4, 5, 6 |
| P8 | 13 | Themes 1, 2, 3, 4, 6 |

# Representative Quotes by Theme

## Theme 1: Diverse Understandings and Perceptions

### Sub-theme 1.1: Pragmatic Views of "Free" Tools

"We need to find some free (software) for them." (P7)

"Free resources are some free tools or free teaching courseware, software, and the like. But my understanding is that open educational resources and free tools or free resources should be two different concepts." (P6)

"Free is not very important to us; if a tool is good or I need to use it, I am willing to pay." (P4)

### Sub-theme 1.2: Nuanced Understanding of Openness

"It should be said that there is an opportunity to achieve open access or open modification. That is, it can open the source code for you to modify." (P6)

### Sub-theme 1.3: Limited Awareness of OEP

"I am not very clear, for students, how I can apply it in my classroom, (in this regard) I am still not very clear." (P8)

"Regarding what open learning resources are... I am not very clear about the definition." (P4)

"I am not very familiar with what this definition is." (P3)

## Theme 2: Current Adoption and Driving Factors

### Sub-theme 2.1: Widespread Use of Interactive Free Tools

"Mainly the most basic Kahoot, as well as Edpuzzle and Nearpod, these are the tools we commonly use." (P6)

"For example, we play Kahoot, it is quite useful. It was commonly used during the pandemic." (P1)

"We use commercial software more than open source software... for example, instant polling on Zoom, or some Google Forms on Google." (P2)

### Sub-theme 2.2: Centrality of the LMS

"We have tried to teach students to use Moodle. Because our students need to go out for teaching practice." (P6)

"Mainly notes I write myself and the functions on OLE, such as submitting assignments and voting." (P3)

### Sub-theme 2.3: Discipline-Specific Adoption

"Fortunately, MatLab has a free alternative, which is GNU software that can be found and used to achieve similar simulation effects." (P5)

"There is VR, MRA, and simulation. There are many different projects underway, and we have a group of colleagues who specialize in developing these teaching materials." (P1)

"Blended learning mode, applied in one of the chemistry courses, dividing experiments into different parts to teach students one by one." (P2)

### Sub-theme 2.4: Key Drivers

"It may help them understand the learning content... some audiovisual media make it easier to understand these concepts." (P5)

"I think students have actually changed now. At that time, I felt that some concepts that were difficult to explain could be visualized for students through these resources, technology, or other methods." (P1)

## Theme 3: Benefits and Opportunities

### Sub-theme 3.1: Enhanced Student Engagement

"It can help consolidate their knowledge. Many times we may not have assessments, and (Edpuzzle) can provide appropriate assessments in real-time to let students know what they are familiar with or unfamiliar with." (P6)

"The students' responses and feedback were very good. This result also moved us, and then we published an article about the plasticity of this blended learning." (P2)

### Sub-theme 3.2: Increased Access to Resources

"One benefit of this open education is that it can reduce educational inequality. For example, students in areas with poorer education can also enjoy free courses provided by some good schools abroad." (P8)

"Before there were open resources, children had less exposure to these things. Now it is easier to access these resources, and more students can benefit." (P3)

### Sub-theme 3.3: Flexibility and Personalization

"We need to provide students with some self-learning resources... if you want to learn these concepts, please watch this video online." (P5)

"Using external resources and tools in our teaching is certainly good, because when we were studying, we did not have so many channels." (P1)

## Theme 4: Challenges and Barriers

### Sub-theme 4.1: OER Quality and Discovery

"The difficulty is that teachers have to find and screen, try each video one by one, to see which videos explain clearly and can be kept." (P5)

"Free ones are rare because we worry that the quality of free teaching tools may be poor." (P1)

"Teachers need time and sufficient resources to apply open educational resources. Finding suitable resources online actually requires considerable investment from teachers." (P2)

### Sub-theme 4.2: Technical Barriers

"Insufficient support. If our Moodle server crashes now, we have to solve the problem ourselves." (P6)

"Commercial software is more powerful in functionality, or easier to use." (P5)

"If I want to do better, I believe that in addition to my current work, I need to invest more time... I need to queue up." (P1)

### Sub-theme 4.3: Time Constraints

"I am one of those very busy people, we really may not have that much time for training." (P7)

"I believe the school should have relevant training, but I am too busy and have no time to attend." (P3)

"Teachers may already be teaching many things, and they may not be willing to spend more time looking at an open source software." (P4)

### Sub-theme 4.4: Institutional Factors

"It is difficult to use open teaching for a 5-credit distance course, not that it cannot be done, but it is difficult because the layout is all fixed." (P7)

"The medical council is quite conservative and does not encourage many new courses." (P1)

"It would be great to have a policy, and if subjects clearly require the inclusion of open tools." (P4)

### Sub-theme 4.5: Digital Literacy

"Some colleagues teaching early childhood education here did not even know how to use YouTube." (P7)

"For some older teachers, I think they may not want to change or learn new things." (P1)

## Theme 5: Evolving Teacher Roles and Professional Development

### Sub-theme 5.1: Shift to Facilitator/Designer

"I am a facilitator, seeing what problems they have." (P7)

"I think I am a teacher, teaching them knowledge, but more importantly, I think I am a tour guide on a tour bus, leading passengers to different attractions." (P2)

"The teacher himself is a user. Because he needs to use these open educational resources to teach students, so he is actually also a student." (P1)

### Sub-theme 5.2: Need for Holistic Professional Development

"Teachers need to keep updating, otherwise they will be replaced by AI." (P5)

"Now, whether it is teachers, doctors, or any industry, everyone needs to learn new things." (P2)

"It would be best to have more training to teach and train teachers on how to use these open software. Some formal training for teachers to let them know more." (P3)

### Sub-theme 5.3: Valuing Peer Learning

"I work with (P6)next door every day researching how to use these software. So it is not very difficult for me, and I can find (P6), who knows many different resources." (P7)

"Colleagues also attend some training classes and share what they have learned with other colleagues." (P1)

## Theme 6: Expectations and Outlook

### Sub-theme 6.1: Improved Resource Quality

"If there is a summary, for example, introducing what tools can do these things. Because teachers may not know, give us this summary." (P8)

"These resources will definitely be used more, there is no reason for them to decrease. So the competition in content and quality will become more intense." (P3)

### Sub-theme 6.2: Stronger Institutional Strategy

"University support, the most important is university support... if the government is very committed to promoting this, the schools will definitely follow." (P7)

"It would be great to have a policy, and if subjects clearly require the inclusion of open tools." (P4)

"I think the university actually needs to explain more about what open learning or flexible teaching means." (P1)

### Sub-theme 6.3: AI Integration Potential

"I think in the future it should be integrated with AI models. More and more companies will develop these AI software for students to use." (P5)

"I think the future development of open education will definitely be digital. And people can see a lot of information anywhere, anytime, regardless of where they are." (P2)

### Sub-theme 6.4: Openness as Trend

"The world should become more and more open... openness and sharing is the trend and direction of this world, and I think education is the same." (P7)

"I think open education will definitely be important in the future. My son's education is already different from our era." (P1)

# Data Saturation Evidence

| Theme | Evidence of Saturation | Participants Contributing |
| --- | --- | --- |
| Theme 1 | Understanding of OER discussed by 6/8 participants; no new conceptualizations emerged after P6 | P1, P3, P4, P6, P7, P8 |
| Theme 2 | Tool usage patterns consistent across all 8 participants; YouTube and interactive tools mentioned by 7/8 | P1, P2, P3, P4, P5, P6, P7, P8 |
| Theme 3 | Benefits consistently identified by 6/8 participants; engagement and access themes repeated | P1, P2, P3, P5, P7, P8 |
| Theme 4 | Challenges highly consistent; time constraints and technical support mentioned by all 8 participants | P1, P2, P3, P4, P5, P6, P7, P8 |
| Theme 5 | Role evolution discussed by 7/8 participants with consistent patterns | P1, P2, P3, P4, P5, P6, P7 |
| Theme 6 | Future expectations converged on AI, policy support, and community building across 7/8 participants | P1, P2, P4, P5, P6, P7, P8 |
